# The multimorbidity of hypertension and osteoarthritis and relation with sleep quality and hyperlipemia/hyperglycemia in China’s rural population

**DOI:** 10.1038/s41598-021-96523-0

**Published:** 2021-08-23

**Authors:** Li Ran, Qi Chen, Jingyi Zhang, Xinlong Tu, Xiaodong Tan, Yuting Zhang

**Affiliations:** 1 Department of Occupational and Environmental Health, School of Health Sciences, Wuhan University, 115 Donghu Road, Wuchang District, Wuhan, 430071 People’s Republic of China; 2 School of Nurse, Wuchang University of Technology, Wuhan, 430223 People’s Republic of China; 3 School of Nursing, Shenzhen University, Shenzhen, 518037 People’s Republic of China

**Keywords:** Ageing, Hypertension, Risk factors

## Abstract

Hypertension (HTN) and osteoarthritis (OA) are frequent in middle-aged and elderly people, and the co-occurrence of these two diseases is common. However, the pathogenesis of the multimorbidity of both diseases and the relation with sleep quality, hyperlipemia, and hyperglycemia is unclear. We conducted a cross-sectional study to make sense of the multimorbidity of HTN and OA and the relation with sleep quality, hyperlipemia, and hyperglycemia. The relation between sleep quality and OA and its joint effect with hyperlipemia or hyperglycemia was evaluated with logistic regression models. The additive interaction was assessed with the relative excess risk due to interaction (REEI), the attributable proportion (AP), and the synergy index (S). According to this research in a remote rural area, approximately 34.2% of HTN patients are accompanied with OA and 49.1% are suffering poor sleep. Both hyperlipemia/hyperglycemia and sleep quality were related to OA prevalence with crude ORs of 1.43 (95% CI 1.014–2.029) and 1.89 (95% CI 1.411–2.519, *P* < 0.001) respectively. An observed additive effect was found greater than the sum of the effects of sleep quality and hyperlipemia/hyperglycemia posed on OA prevalence alone. This additive interaction was observed in females (OR = 3.19, 95% CI 1.945–5.237) as well as males ≥ 65 years old (OR = 2.78, 95% CI 1.693–4.557), with RERI, AP, and S significant. Therefore, poor sleep and hyperlipemia/hyperglycemia are associated with OA, and further studies on the additive interaction among females and males ≥ 65 are warranted.

## Introduction

Age-associated multimorbidity has been a common but complex issue over the world, which means the coexistence of several chronic diseases in a person for a long period^1,2^. Due to the high prevalence, every year multimorbidity caused a major burden on personal health and for social cost in regions including Asia^3,4^.

Based on the researches to date, about half of chronic disease patients had comorbidities, and multimorbidity was common to see in patients with hypertension (HTN) and osteoarthritis (OA). According to a South African primary healthcare study, 23.70% of OA patients and 12.00% of HTN patients have comorbidities^5^. As a developing country with a huge population, China shows a similar situation in co-existing diseases in 28.78% and 21.29% of patients respectively with OA and HTN^3^.

Among the many comorbidities, the multimorbidity of HTN and OA occurred frequently^6,7^. Epidemiological studies have found that HTN patients significantly have a 1.394 times higher risk of OA, with the comorbidity rate ranged from 20 to 80% in both developed and developing countries^8^. Although OA was not the most common comorbid disease in HTN, the risk—a prevalent pathology of cartilage in joints, a leading cause of disability, a primary factor of decreased life quality, as well as a high rate of hospitalization—makes it an important topic and worthy to study especially since corresponding research among the Chinese population is limited^4,9,10^. Further, learning more about OA does help in minimizing the adverse impact on blood pressure control in hypertensive patients.

Following more reports on OA and cardiovascular risk factors, interest in relevant mechanisms and factors between OA and HTN is increasing. Factors associated with this multimorbidity were complex and various, mainly including age, sex, and quality of life^11,12^, and related population-based studies seem to favor the etiology of ischemia and lipid metabolism with HTN. They thought HTN might decrease peripheral blood flow, resulting in subchondral ischemia and osteocyte cell death^13^; deregulated lipid metabolism, a possible strong OA determinant, might induce ectopic lipid deposition, initiating OA development^14^. However, it has not been adequately addressed and no unified conclusion has been drawn.

Adding to the confusion, more sleep disorders, shorter sleep duration, and poorer sleep quality are likely to the complaint of OA, and have been demonstrated to be a risk of HTN. For example, hypertension risk might be increased by sleep disorders, especially sleep deprivation^15^ and inappropriate sleep duration^16^, while some studies suggested that sleep disturbance might increase the occurrence of OA^17,18^, as does the finding might be associated with HTN^19^. On the contrary, another study pointed that the presence of chronic OA has the potential to increase the risk for incurring sleep disturbances^20^. It is easy to see there is a limited understanding of the link between sleep quality and OA up to now.

As possible factors associated with OA, the interaction effect between metabolic syndrome (including hyperglycemia and hyperlipidemia) and sleep has not been specifically studied with OA prevalence as an endpoint, much fewer patients with HTN out of that. Therefore, if there is an interaction between sleep and hyperglycemia/hyperlipidemia to the prevalence of OA, then it brings a sense of enlightenment to implement preventive behaviors for protection and control. Besides, it may provide a possible recommendation for combinational therapy and nursing for OA and HTN in clinical practices. Consequently, it is necessary to conduct this study to make up the gap.

In summary, sleep quality, lipid metabolism, OA, and HTN are intertwined, very complex, and not fully elucidated. We included hypertensive patients in a poverty-stricken rural area to make clear the OA prevalence and to ascertain the relation of sleep quality and hyperlipidemia/hyperglycemia with the prevalence of OA, and further to confirm the interaction effect between them.

## Materials and methods

### Study population

This cross-sectional study included HTN patients from Xuan’en county in a remote mountainous area of central China. All participants are local inhabitants over 18 years old and volunteered to participate, and they have no obstacle in communication. HTN was defined as systolic blood pressure (SBP) ≥ 140 mmHg or diastolic blood pressure (DBP) ≥ 90 mmHg, or current use of antihypertensive medication. The sample size was estimated with the average prevalence of hypertension in China with the equation: $n={{Z}_{(\alpha /2)}}^{2}\times p\times (1-p)/{\delta }^{2}$, where $\alpha$ is 0.05, $\delta$ is 0.15*p*, and *p* is 30%. To compensate for the non-response rate, the sample was increased by 10% with a final sample size of 685. The study followed the guidelines of the Declaration of Helsinki, and ethical approval and informed consent were obtained.

### Data collection

The current cross-sectional data were collected from April to July 2018. Data collection took place in two stages: in the first stage, the study collected a broad range of information on sociodemographic characteristics (e.g. sex, age, marital status, monthly income, education level, place of residency), health-related conditions (e.g. BP value, height, weight, waist, hist), and doctor-diagnosed or self-reported OA (yes or no), hyperlipemia, and hyperglycemia. The second stage comprised face-to-face interviews with structured questionnaires to make sense of their sleep quality and compliance with hypertension.

BP values were taken three times using a mercury gauge sphygmomanometer. Consecutive BP measurements were taken at 5-min intervals from the right arm by our investigators, and the average was used for analysis. The information on participants’ sleep quality was gathered with the standard Pittsburgh Sleep Quality Index (PSQI). Seven dimensions were used to describe participants’ sleep quality, including subjective sleep quality, sleep latency, sleep duration, habitual sleep efficiency, sleep disturbances, sleep medication, and daytime dysfunction. The total score of the PSQI scale ranges from 0 to 21, with lower scores indicating better sleep quality. Compliance of hypertension was measured using the Compliance of Hypertensive Patients’ scale (CHPS), which contained 14 items. Items were evaluated with a 4-point Likert scale, where a higher score indicated a higher level of compliance.

### Statistical analysis

Continuous variables are described as means ± standard deviations (M ± SD) and median (interquartile range [IQR]), while frequency and percentages are presented for categorical variables. Differences in sample characteristics were assessed with a chi-squared test for categorical variables. Independent-sample *t*-test and Mann–Whitney *U*-test were used for normally distributed and skewed continuous variables.

The independent association between sleep quality and OA, as well as its joint effect (additive interaction) with hyperlipemia or hyperglycemia, were evaluated with logistic regression models after adjusting covariates. Sleep quality was divided into high group (or reference group) if the PQSI score ≤ 6 and low group > 6 scores. Results were illustrated as unadjusted and adjusted odds ratio (OR) and its 95% confidence interval (95% CI). According to Hosmer and Lemeshow^21^, additive interaction was assessed with the relative excess risk due to interaction (REEI), the attributable proportion (AP), and the synergy index (S); the 95% CIs for the former two indexes should not include 0 and the latter one should not include 1. We calculated RERI by using the formula:

$${\text{RR}}_{{{\text{hyperlipemia or hyperglycemia}},{\text{ poor sleep}}}} - {\text{ RR}}_{{{\text{hyperlipemia or hyperglycemia}},{\text{ good sleep}}}} - {\text{ RR}}_{{{\text{no hyperlipemia or hyperglycemia}},{\text{ poor sleep}}}} + {1}.$$

All statistical analyses were performed using STATA version 14.0 (StataCorp. 2015. Stata Statistical Software: Release 14. College Station, TX: StataCorp LP.), and comparisons with *P*-values < 0.05 were considered statistically significant.

### Ethical statement

Ethics approval for this study was granted by the Research Ethics Boards of Wuhan University (project approval code: 2018-1602000-03-02). Informed consent was obtained from all survey participants.

## Results

### Characteristics of study participants

A total of 837 HTN individuals were comprised of 379 (45.3%) males and 458 (54.7%) females, aged from 30 to 96 years with a median of 69. About half (49.5%) participants were of Tujia nationality in this study area. Most participants were characterized with low education level (primary school and illiteracy accounted for 79.7%) and family monthly income (< ¥3000 accounted for 84.2%).

#### According to OA dichotomy

The total sample numbered 286 suffered from OA, accounting for 34.2% of the whole participants. Of which, 11 individuals were self-reported OA with typical symptoms like stiffness, swelling, and deformity, and their sleep quality (the *P*-values for total score and 7 dimensions in PSQI were above 0.10) and all demographic and clinical characteristics (*P*s > 0.10) has good homogeneity with the doctor-diagnosed ones. So, they were involved in the analysis. Table 1 shows the distribution differences in the prevalence of OA by participants’ demographic, clinical, sleep, and compliance characteristics. OA prevalence varies in different ages (*P* = 0.001), sexes (*P* < 0.001), occupations (*P* = 0.023), educations (*P* < 0.001), monthly incomes (*P* = 0.013), sleep quality scores (*P* < 0.001), and several dimensions of PSQI except for sleep duration and sleep medicine.

**Table 1:** Participants’ characteristics according to OA status stratification.

Characteristics	Total (n = 837)	Osteoarthritis		
		Absence (n = 551)	Presence (n = 286)	*P*-value
**Demographic and clinical characteristics**				
Age, y (Md, IQR)	69 (62–74)	68 (60–74)	69 (64–75)	**0.001**
Sex-men, n (%)	379 (45.3)	276 (50.1)	103 (36.0)	**< 0.001**
Ethnic-Tujia, n (%)	414 (49.5)	289 (52.5)	125 (43.7)	0.004
Marriage-married, n (%)	617 (73.7)	411 (74.6)	206 (72.0)	0.882
Place of residency-country, n (%)	783 (93.6)	510 (92.6)	273 (95.5)	0.190
Occupation-famer, n (%)	741 (88.5)	470 (85.3)	271 (94.8)	**0.023**
Education-primary school, n (%)	392 (46.8)	256 (46.5)	136 (47.6)	**< 0.001**
Duration of hypertension-< 1 year, n (%)	333 (39.8)	226 (41.0)	107 (37.4)	0.339
Family monthly income-< 1000 RMB, n (%)	386 (46.1)	249 (45.2)	137 (47.9)	**0.013**
Smoking-never, n (%)	564 (67.4)	363 (65.9)	201 (70.3)	0.253
Alcohol-never, n (%)	563 (67.3)	366 (66.4)	197 (68.9)	0.719
Tea-often, n (%)	313 (37.4)	194 (35.2)	119 (41.6)	0.214
SBP, mmHg (Md, IQR)	138.3 ± 22.9	138.7 ± 23.3	137.4 ± 22.1	0.751
DBP, mmHg (Md, IQR)	84.8 ± 13.0	84.9 ± 12.9	84.6 ± 13.1	0.563
BMI, kg/m^2^ (Md, IQR)	22.8 ± 3.8	22.8 ± 3.9	22.8 ± 3.7	0.524
WHR (Md, IQR)	0.9 ± 0.1	0.9 ± 0.1	0.9 ± 0.1	0.108
**Pittsburgh Sleep Quality Index score**				
Subjective sleep quality (Md, IQR)	1 (1–2)	1 (1–2)	1 (1–2)	**< 0.001**
Sleep latency (Md, IQR)	1 (1–2)	1 (0–2)	1 (0–2)	**< 0.001**
Sleep duration (Md, IQR)	0 (0–1)	0 (0–1)	0 (0–1)	0.099
Habitual sleep efficiency (Md, IQR)	0 (0–0)	0 (0–0)	0 (0–0)	**0.048**
Sleep disturbances (Md, IQR)	1 (1–2)	1 (1–2)	2 (1–2)	**0.001**
Sleep medicine (Md, IQR)	0 (0–0)	0 (0–0)	0 (0–0)	0.058
Daytime dysfunction (Md, IQR)	2 (2–3)	2 (2–3)	3 (2–3)	**0.016**
Total score (Md, IQR)	6 (5–9)	6 (4–9)	7 (5–10)	**< 0.001**
**Compliance of Hypertensive Patients Scale score**				
Intention (Md, IQR)	6 (0–8)	6 (0–8)	6 (0–8)	0.908
Lifestyle (Md, IQR)	6 (0–9)	7 (0–9)	5 (0–8)	0.062
Attitude (Md, IQR)	3 (0–5)	3 (0–5)	3 (0–5)	0.975
Responsibility (Md, IQR)	2 (0–4)	2 (0–4)	2 (0–4)	0.333
Smoking (Md, IQR)	1 (0–1)	1 (0–1)	1 (0–1)	0.960
Medication (Md, IQR)	1 (0–2)	1 (0–2)	1 (0–1)	0.442
Total score (Md, IQR)	23 (0–29)	23 (0–23)	23 (0–28)	0.408

*Md* median, *IQR* interquartile range, *SBP* systolic blood pressure, *DBP* diastolic blood pressure, *BMI* body mass index, *WHR* waist-to-hip ratio.

Significant differences (*P* < 0.05) are highlighted in bold.

#### According to hyperlipemia/hyperglycemia and sleep quality

Among the HTN individuals recruited in this study, 169 (20.2%) displayed symptoms of hyperlipemia or hyperglycemia while 411 (49.1%) manifested with low sleep quality. As seen in Table 2, hyperlipemia or hyperglycemia status were found different in HTN duration (*P* < 0.001), smoking (*P* = 0.002), alcohol (*P* = 0.001), all items in CHPS (*Ps* < 0.05), and several dimensions in PSQI (except for sleep latency, sleep duration, and sleep medicine). When categorizing the total participants according to their sleep quality, significant differences were in sex (*P* = 0.002), marriage (*P* = 0.027), occupation (*P* = 0.007), education (*P* = 0.001), HTN duration (*P* = 0.002), tea-drinking (*P* = 0.012), SBP (*P* = 0.034), and all dimensions in PSQI (*Ps* < 0.01) and CHPS (*Ps* < 0.05).

**Table 2:** Participants’ characteristics in hyperlipemia/hyperglycemia and sleep quality.

Characteristics	Hyperlipemia or hyperglycemia			Sleep quality		
	Absence (n = 668)	Presence (n = 169)	*P*-value	High (n = 426)	Low (n = 411)	*P*-value
**Demographic and clinical characteristics**						
Age, y (Md, IQR)	69 (61–74)	69 (63.5–75)	0.242	69 (62–74)	69 (63–74)	0.805
Sex-men, n (%)	307 (46.0)	72(42.6)	0.434	215 (50.5)	164 (39.9)	**0.002**
Ethnic-Tujia, n (%)	334 (50.0)	80 (47.3)	0.586	202 (47.4)	212 (51.6)	0.642
Marriage-married, n (%)	493 (73.8)	124 (73.4)	0.829	329 (77.2)	288 (70.1)	**0.027**
Place of residency-country, n (%)	625 (93.6)	158 (93.5)	0.214	393 (92.3)	390 (94.9)	0.179
Occupation-famer, n (%)	594 (88.9)	147 (87.0)	0.205	196 (46.0)	196 (87.2)	**0.007**
Education-primary school, n (%)	311 (46.6)	81 (47.9)	0.095	37 (49.3)	44 (47.7)	**0.001**
Duration of hypertension-< 1 year, n (%)	285 (42.7)	48 (28.4)	**< 0.001**	193 (45.3)	140 (34.1)	**0.002**
Family monthly income-< 1000 RMB, n (%)	319 (47.8)	67 (39.6)	0.306	188 (44.1)	198 (41.5)	0.077
Smoking-never, n (%)	443 (66.3)	121 (71.6)	**0.002**	59 (78.7)	62 (48.2)	0.688
Alcohol-never, n (%)	442 (66.2)	121 (71.6)	**0.001**	287 (67.4)	277 (67.4)	0.142
Tea-often, n (%)	243 (36.4)	70 (41.4)	0.241	368 (86.4)	373 (90.8)	**0.012**
SBP, mmHg (Md, IQR)	138.3 ± 24.0	138.1 ± 17.7	0.717	137.2 ± 23.6	139.4 ± 22.1	**0.034**
DBP, mmHg (Md, IQR)	85.0 ± 13.4	83.8 ± 11.2	0.435	84.8 ± 12.6	84.8 ± 13.4	0.884
BMI, kg/m^2^ (Md, IQR)	22.7 ± 3.8	23.0 ± 4.1	0.456	22.8 ± 3.8	22.8 ± 3.9	0.635
WHR (Md, IQR)	0.9 ± 0.1	0.9 ± 0.1	0.065	0.9 ± 0.1	0.9 ± 0.1	0.066
**Pittsburgh Sleep Quality Index score**						
Subjective sleep quality (Md, IQR)	1 (1–2)	1 (1–2)	**0.008**	1 (0–1)	2 (1–2)	**< 0.001**
Sleep latency (Md, IQR)	1 (0–2)	1 (0–2)	0.122	0 (0–1)	2 (1–2)	**< 0.001**
Sleep duration (Md, IQR)	0 (0–1)	0 (0–1)	0.648	0 (0–0)	1 (0–1)	**< 0.001**
Habitual sleep efficiency (Md, IQR)	0 (0–0)	0 (0–1)	**0.010**	0 (0–0)	0 (0–1)	**< 0.001**
Sleep disturbances (Md, IQR)	1 (1–2)	2 (1–2)	**0.001**	1 (1–2)	2 (1–2)	**< 0.001**
Sleep Medicine (Md, IQR)	0 (0–0)	0 (0–0)	0.633	0 (0–0)	0 (0–0)	**0.006**
Daytime dysfunction (Md, IQR)	2 (2–3)	3 (2–3)	**0.006**	2 (1–2)	3 (3–3)	**< 0.001**
Total score (Md, IQR)	6 (4–9)	7 (5–10)	**0.001**	5 (4–6)	9 (8–11)	**< 0.001**
**Compliance of Hypertensive Patients Scale score**						
Intention (M ± SD)	6 (0–8)	6 (4–9)	**0.001**	5 (0–7)	6 (0–9)	**< 0.001**
Lifestyle (M ± SD)	6 (0–9)	7 (3–9)	**< 0.001**	5 (0–9)	7 (0–9)	**0.001**
Attitude (M ± SD)	3 (0–5)	4 (3–5)	**0.008**	3 (0–5)	4 (0–5)	**< 0.001**
Responsibility (M ± SD)	2 (0–4)	2 (2–5)	**< 0.001**	2 (0–4)	2 (0–4)	**< 0.001**
Smoking (M ± SD)	1 (0–1)	1 (1–1)	**0.006**	1 (0–1)	1 (0–1)	**0.011**
Medication (M ± SD)	1 (0–2)	1 (1–2)	**0.024**	1 (0–2)	1 (0–2)	**0.015**
Total score (M ± SD)	22 (0–29)	25 (19–29.5)	**0.008**	21 (0–28)	25 (0–31)	**< 0.001**

*Md* median, *IQR* interquartile range, *SBP* systolic blood pressure, *DBP* diastolic blood pressure, *BMI* body mass index, *WHR* waist-to-hip ratio.

Significant differences (*P* < 0.05) are highlighted in bold.

### Association between PSQI and osteoarthritis

The association between PSQI and OA is shown in Table 3. Exposure to hyperlipemia/hyperglycemia and poor sleep quality were both associated with the prevalence of OA in unadjusted and adjusted models. For the participants who suffered hyperlipemia or hyperglycemia, the crude OR was 1.43 (95% CI 1.014–2.029, *P* = 0.042). Results were also significant in adjusted models (for model 1: 1.83, 95% CI 1.355–2.477, *P* < 0.001; for model 2: 1.52, 95% CI 1.049–2.214, *P* = 0.027).

**Table 3:** Associations of hyperlipemia/hyperglycemia and PSQI with osteoarthritis.

Predictors	Odds ratio (95% CI)		
	Unadjusted model	Adjusted model 1	Adjusted model 2
**Hyperlipemia or hyperglycemia**			
No	Reference	Reference	Reference
Yes	1.43 (1.014–2.029)	1.83 (1.355–2.477)	1.52 (1.049–2.214)
*P* value for trend	**0.042**	**< 0.001**	**0.027**
**Sleep quality (score)**			
High (≤ 6)	Reference	Reference	Reference
Low (7–21)	1.89 (1.411–2.519)	1.79 (1.333–2.415)	1.83 (1.352–2.476)
*P* value for trend	**< 0.001**	**< 0.001**	**< 0.001**

*95% CI* 95% confidence interval. The prevalence of osteoarthritis was regarded as a dependent variable. Adjusted model 1 was adjusted for sex, age, and occupation; Adjusted model 2 was adjusted for variables included in model 1 and variables in Compliance.

Significant differences (*P* < 0.05) are highlighted in bold.

Similar in HTN patients with poor sleep quality, the crude OR was 1.89 (95% CI 1.411–2.519, *P* < 0.001). The OR was 1.79 (95% CI 1.333–2.415, *P* < 0.001) after adjusting for sex, age, and occupation; the OR was 1.83 (95% CI 1.352–2.476, *P* < 0.001) adjusting for additional variables in CHPS.

### Joint effects of sleep quality and hyperlipemia/hyperglycemia

Table 4 shows the additive effect of sleep quality with hyperlipemia/hyperglycemia on the prevalence of OA. In all participants, when compared with the reference group, the OR for individuals in low sleep quality and hyperlipemia/hyperglycemia was 2.52 (95% CI 1.377–4.609). The RERI, AP, and S were respectively 1.16 (95% CI − 0.099 to 2.413), 0.43 (95% CI 0.095–0.771), and 3.24 (95% CI 0.681–15.446) with no significance. Similar insignificant results approached in males, and people ≥ 65 or < 65.

**Table 4:** Additive effect of PSQI with hyperlipemia/hyperglycemia on osteoarthritis.

	Odds ratio (95% CI)	Evaluation index (95% CI)		
		RERI	AP	S
**All participants (n = 837)**				
Low sleep quality	**1.73 (1.257–2.382)**	0.77 (− 0.795 to 2.342)	0.31 (− 0.178 to 0.792)	2.04 (0.502–8.274)
Hyperlipemia/hyperglycemia	1.02 (0.541–1.908)			
Low sleep quality + hyperlipemia/hyperglycemia	**2.52 (1.377–4.609)**			
**Male (n = 379)**				
Low sleep quality	1.59 (0.942–2.697)	0.49 (− 1.260 to 2.244)	0.23 (− 0.486 to 0.947)	1.77 (0.203–15.391)
Hyperlipemia/hyperglycemia	1.05 (0.451–2.424)			
Low sleep quality + hyperlipemia/hyperglycemia	**2.13 (1.000–4.546)**			
**Female (n = 458)**				
Low sleep quality	**1.68 (1.200–2.340)**	**1.40 (0.001–2.798)**	**0.44 (0.173–0.704)**	**2.77 (1.164–6.574)**
Hyperlipemia/hyperglycemia	**1.12 (1.057–1.180)**			
Low sleep quality + hyperlipemia/hyperglycemia	**3.19 (1.945–5.237)**			
**Age ≥ 65 (n = 668)**				
Low sleep quality	**1.54 (1.063–2.226)**	1.15 (− 0.318 to 2.610)	**0.42 (0.031–0.801)**	2.89 (0.614–13.581)
Hyperlipemia/hyperglycemia	1.07 (0.579–1.975)			
Low sleep quality + hyperlipemia/hyperglycemia	**2.75 (1.607–4.718)**			
**Age < 65 (n = 169)**				
Low sleep quality	1.97 (0.835–4.664)	2.60 (− 1.908 to 7.100)	**0.67 (0.150–1.181)**	9.55 (0.022–4119.073)
Hyperlipemia/hyperglycemia	0.33 (0.038–2.840)			
Low sleep quality + hyperlipemia/hyperglycemia	**3.90 (1.107–13.740)**			
**Male + Age ≥ 65 (n = 315)**				
Low sleep quality	**1.53 (1.135–2.074)**	**1.14 (0.053–2.331)**	**0.41 (0.143–0.677)**	**2.78 (1.118–6.930)**
Hyperlipemia/hyperglycemia	**1.11 (1.046–1.167)**			
Low sleep quality + hyperlipemia/hyperglycemia	**2.78 (1.693–4.557)**			
**Female + Age ≥ 65 (n = 353)**				
Low sleep quality	**1.63 (1.389–1.904)**	**1.81 (0.790–2.827)**	**0.50 (0.279–0.721)**	**3.24 (1.259–8.329)**
Hyperlipemia/hyperglycemia	**1.18 (1.083–1.291)**			
Low sleep quality + hyperlipemia/hyperglycemia	**3.62 (2.859–4.575)**			

*95% CI* 95% confidence interval, *RERI* relative excess risk due to interaction, *AP* attributable proportion, *S* synergy index.

Significant differences (*P* < 0.05) are highlighted in bold. Models were adjusted for age, sex, monthly income, education level, occupation, smoking, alcohol intake frequency, tea intake frequency, and duration of hypertension.

**Figure 1:**
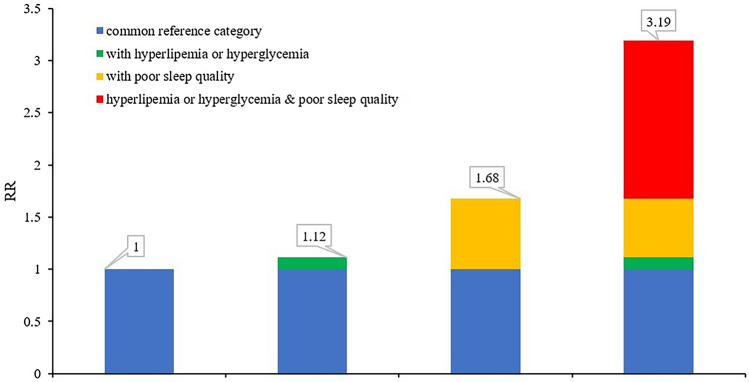
An additive effect of PSQI and hyperlipemia/hyperglycemia for females.

For females, the observed additive effect was greater than the sum of the effects of sleep quality and hyperlipemia/hyperglycemia alone: the OR of poor sleep quality was 1.68 (95% CI 1.200–2.340), hyperlipemia or hyperglycemia was 1.12 (95% CI 1.057–1.180), and poor sleep quality and hyperlipemia/hyperglycemia was 3.19 (95% CI 1.945–5.237). RERI (1.40, 95% CI 0.001–2.798), AP (0.44, 95% CI 0.173–0.704), and S (2.77, 95% CI 1.164–6.574) were statistically significant. (See details in Table 4 and Fig. 1).

**Figure 2:**
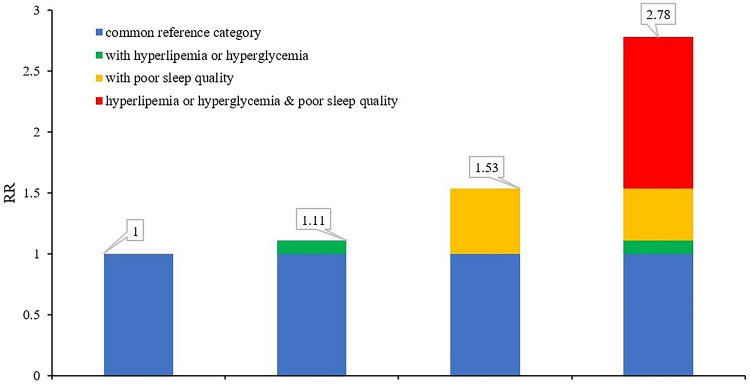
An additive effect of PSQI and hyperlipemia/hyperglycemia for males ≥ 65.

**Figure 3:**
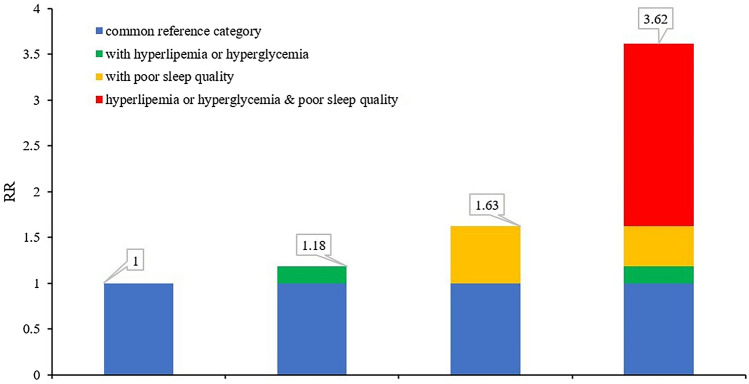
An additive effect of PSQI and hyperlipemia/hyperglycemia for females ≥ 65.

We further conducted a sex-stratification analysis on top of age-stratification, and a positive interaction was noted both in males aged ≥ 65 and females aged ≥ 65. The degree of synergy for males ≥ 65 can be seen in Table 4 and Fig. 2: the OR of poor sleep quality, hyperlipemia or hyperglycemia, and poor sleep quality with hyperlipemia/hyperglycemia was respectively 1.53 (95% CI 1.135–2.074), 1.11 (95% CI 1.046–1.167), and 2.78 (95% CI 1.693–4.557). For females aged ≥ 65 years, the OR of poor sleep quality, hyperlipemia or hyperglycemia, and poor sleep quality and hyperlipemia/hyperglycemia was 1.63 (95% CI 1.389–1.904), 1.18 (95% CI 1.083–1.291), and 3.62 (95% CI 2.859–4.575). RERI, AP, and S were statistically significant. (See details in Table 4 and Fig. 3).

## Discussion

Analysis of the data from Xuan’en area shows a 34.2% prevalence of OA in hypertensive individuals, indicating a not optimistic situation. Approximately 20.2% of HTN patients have hyperlipemia or hyperglycemia, and 49.1% are suffering poor sleep. In addition, poor sleep quality demonstrates a positive correlation with OA, and it also shows an additive effect with hyperlipemia or hyperglycemia among the males ≥ 65 years and females, especially those ≥ 65.

This investigation is a preliminary attempt to explore sleep quality and its additive relation with the multimorbidity of HTN and OA in a resource-limited mountainous area, central China. Epidemiological researches reveal that sleep problems are frequent in all populations and more than half of the elderly older than 65 suffer from it due to chronic diseases or external environments^22^. Notably, in this study, poor sleep showed a more pronounced tendency in HTN patients with OA, including subjective sleep, sleep latency, habitual sleep efficiency, sleep disturbances, daytime dysfunction, and total score, even after adjusting for covariates. This was similar to the findings from Regina et al.^23,24^. Besides, the multimorbidity of OA and HTN found here is much higher than China's overall level of 14.00%, according to China Health and Retirement Longitudinal Study^3^. Because Xuan’en is a poverty-stricken area with a large number of young people leaving for urban, complicated and diverse chronic diseases of the elderly as well as low-income level, inadequate health literacy, and the absence of adequate medical services undoubtedly constitute a huge threat to life health and quality. Given that rural elderly is still inferior in physical health to their counterparts in urban areas^25^, more medical resources and fundamental changes are advocated preferential to these regions. Therefore, more economical and convenient screening and referral to primary care is our most urgent concern.

Up to now, the bi-directional relation between sleep and OA has been a major point of discussion in publications concerning this domain, but the possible mechanism is not completely clear yet. A previous national study found a U-shape curve between the prevalence of OA and sleep duration with a nadir in the 7–8 h sleep category^26^. A case–control study additionally proved that sleep disorder was associated to a significant extent with higher odds of developing OA^27^. Based on the above ideas, we hypothesized that there would be direct or indirect associations between OA and sleep quality, and verified it in this study. Although accurate causal relation cannot be inferred due to the limitation of a cross-sectional study, it’s suggested that HTN patients with OA could be regularly screened for sleep disturbance with PSQI as a way to relieve the shortage of medical services and medicine. PSQI is a generic scale for measuring the quality and patterns of sleep in adults, and it could be of use for the management of OA and HTN patients^28^. It was shown to have strong reliability and validity with a sensitivity of 98.7 and specificity of 84.4 to differentiate sleep disturbances^29^. For the result in our additive interaction analysis, HTN patients aged ≥ 65 years, especially with hyperlipemia or hyperglycemia, are appropriate to use this self-reported instrument to warn of OA development or exacerbation in remote areas. However, to ensure the stability and feasibility of the results, it awaits to be confirmed by further researches.

Currently, the interactions between HTN, metabolism, sleep disorders, and OA are intertwined and complicated. The links between them are not fully elucidated, but a longitudinal study illustrated that poor sleep quality is associated with cardiovascular diseases (CVDs), in particular HTN, starting with microvascular dysfunction^30^. Other studies confirmed that increased BP is attributed to the metabolic syndrome-related mechanism due to poor sleep^31,32^. Metabolic syndrome, especially abnormal lipid metabolism, also contributed to the formation and development of OA^14^. All these might provide an idea for explaining a higher prevalence of OA among HTN accompanied by poor sleep quality and hyperlipemia/hyperglycemia in this research. The link between lipid metabolism, sleep quality, and HTN further strengthens the importance of fostering optimal sleep in preventing or ameliorating the development of OA.

Furthermore, it is novel and attractive in finding a significant additive effect among females after adjusting for age. So far, the only evidence for a gender-age difference has been through several works of literature, the overall prevalence of OA and CVDs (including hypertension, hyperlipemia et al.) was separately higher in females compared to males over the age of 50^33,34^. The explanation for the higher multimorbidity of HTN and OA in females is associated with the estrogen level, as it correspondingly decreased after peak menopausal age^35^. That, plus the fact that females are more vulnerable to poor sleep than males, may explain why the joint effect of HTN, sleep quality, and hyperlipemia/hyperglycemia is more notable in females.

To our knowledge, this is the first study to report the additive interaction between sleep and hyperlipemia/hyperglycemia linked with OA prevalence. Nonetheless, several limitations should be addressed. Due to the study design of a cross-sectional study, no causal relationship between sleep quality and the prevalence of OA can be inferred. Therefore, it calls for more rigorous designs to identify predictive factors of sleep impairment in OA. Also, the absence of evaluation indexes that include the intensity of pain, quality of life, and condition of medication makes it difficult to clarify disease outcomes and turnover for HTN patients with OA. Because of a few studies and small samples, we are inspired to go into much depth on this topic in the future with more large-scale studies and multi-regional cooperations.

## Conclusion

Poor sleep quality is common and troublesome among people with HTN complicated with OA, and it shows a positive correlation with the prevalence of OA. The additive interaction between HTN, hyperlipemia or hyperglycemia, and sleep quality was observed in females and males ≥ 65, but it was complex and not fully elucidated. Further studies on the links are warranted.

## Data Availability

The datasets generated for this research are available on request to the corresponding author.

## Abbreviations

HTN: Hypertension
OA: Osteoarthritis
SBP: Systolic blood pressure
DBP: Diastolic blood pressure
CHPS: Compliance of Hypertensive Patients’ scale
PSQI: Pittsburgh Sleep Quality Index
OR: Odds ratio
REEI: Relative excess risk due to interaction
AP: Attributable proportion
S: Synergy index
CVD: Cardiovascular disease
